# Budd–Chiari Syndrome Due to Protein C Deficiency: A Rare Disorder to cause Chronic Liver Disease

**DOI:** 10.5005/jp-journals-10018-1199

**Published:** 2016-12-01

**Authors:** Rukshana Begum, Mamun Al Mahtab, Ayub Al Mamun, Ahmed Lutful Moben, Sharker Mohammad Shahadat Hossain, Dulal Chandra Das, Debraj Malakar, Harun Or Rashid, Partho Protim Roy, Salimur Rahman

**Affiliations:** 1 Department of Hepatology, Bangabandhu Sheikh Mujib Medical University, Dhaka, Bangladesh

**Keywords:** Budd–Chiari Syndrome, Protein C deficiency, Rare disorder.

## Abstract

The Budd–Chiari syndrome (BCS) is a rare disorder due to chronic liver disease (CLD), which is caused by the obstruction of hepatic venous outflow that can be located at any place from the small hepatic venules up to the entrance of the inferior vena cava (IVC) into the right atrium. Among the causes of BCS, the rarer one is coagulation factor deficiencies. Here, we report a case of BCS associated with deficiency of protein C resulting in thrombus in IVC. The patient was a 50-year-old male, who had been suffering from recurrent abdominal and leg swelling for a long period of 7 years. He was evaluated thoroughly, and other causes of liver cirrhosis were excluded.

**How to cite this article:**

Begum R, Al Mahtab M, Al Mamun A, Moben AL, Hossain SMS, Das DC, Malakar D, Rashid HO, Roy PP, Rahman S. Budd–Chiari Syndrome Due to Protein C Deficiency: A Rare Disorder to cause Chronic Liver Disease. Euroasian J Hepato-Gastroenterol 2016;6(2):194-197.

## INTRODUCTION

Budd–Chiari syndrome (BCS) is by consensus characterized by obstruction of the hepatic venous outflow tract, regardless of the mechanism and level of obstruction, but excluding cardiac and pericardial disease and sinusoidal obstruction syndrome.^1^ In Western countries, BCS usually occurs due to thrombosis of hepatic veins, but in Asia, it is most frequently seen due to obstruction of the inferior vena cava (IVC) or combined IVC/hepatic veins block.^2^ The etiology of BCS can be diverse. The most frequently responsible disease for BCS is myeloproliferative neoplasms (39%), JAK2 mutation (29%), antiphospholipid syndrome (APLS) (25%), paroxysmal nocturnal hemoglobinuria (PNH) (19%), protein C deficiency (4%), and protein S deficiency (3%).^3^ So protein C deficiency as a cause of BCS is not so common. In this report, we present a case of BCS caused by IVC obstruction as a result of protein C deficiency.

## CASE REPORT

**Figs 1A and B:**
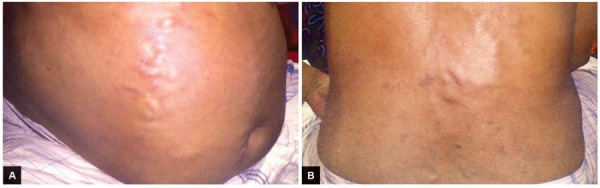
Engorged rope-like tortuous veins from front and back of abdomen

A 50-year-old male patient was admitted to Hepatology Department, Bangabandhu Sheikh Mujib Medical University (BSMMU), Dhaka, Bangladesh, with complaints of recurrent abdominal and leg swelling with scrotal swelling for 7 years, visible rope-like engorged vein over abdomen and back for 3 years, and generalized weakness and anorexia for 6 months. He gave no personal or family history of any venous thromboembolic events. He gave no history of alcohol or tobacco consumption or exposure to relevant drugs and hepatotoxic chemicals. Physical examination demonstrated hepatic facies with mild anemia, gynecomastia and bilateral ankle edema. On per abdominal examination, there was rope-like, tortuous engorgement of the superficial vessels on the anterior abdominal wall, chest wall, and back with flow toward the superior vena cava (Figs 1A and B). Liver was palpable with left lobe enlargements, which was firm, nontender, smooth surface, and no bruit. Spleen was also palpable, and there were mild ascites, scrotal edema, with soft nontender testis. Other physical examination findings were unremarkable.

The laboratory findings were as follows: Aspartate transaminase 31 IU/L (normal range 14–63 IU/L), alkaline phosphatase 107 IU/L (normal range 60–240 IU/L), serum albumin 4.8 gm/dL (normal range 3.8–5.4 gm/dL), total bilirubin 1.6 mg/dL (normal range 0–1.1 mg/dL), erythrocyte count 4.9 × 10^12^/L (normal range 4.5–5.5 × 10^12^/L), hemoglobin concentration 14.3 gm/dl (normal range 13–17 gm/dL), leukocyte count 6 × 10^9^/μL (normal range 4–11 × 10^9^/L), platelet count 200 × 10^9^/L (normal range 150–400 × 10^9^/L), prothrombin time 17.2 seconds (normal range 11.0–15.0 seconds), international normalized ratio (INR) 1.44 (normal range 0.8–1.2), and serum AFP 3.7 ng/mL. Ascitic fluid study revealed protein 2.8 gm/dL, albumin 2.6 gm/dL, Serum ascites albumin gradient (SAAG) was 2.2, Acid fast bacilli (AFB) and Gram-stain negative. Serological tests were negative for hepatitis A, B, C, and E. Serum ferritin and ceruloplasmin are within normal limit.

**Fig. 2:**
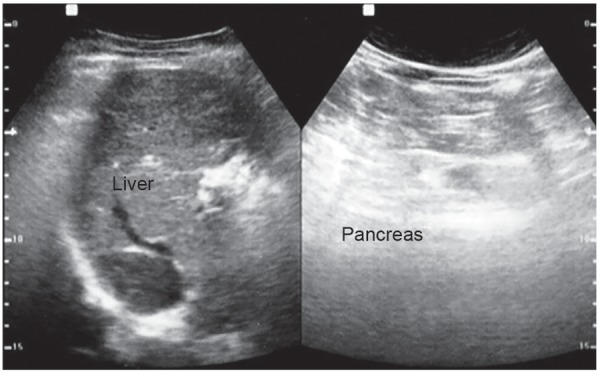
Ultrasonography of hepato-biliray system (HBS)

**Fig. 3:**
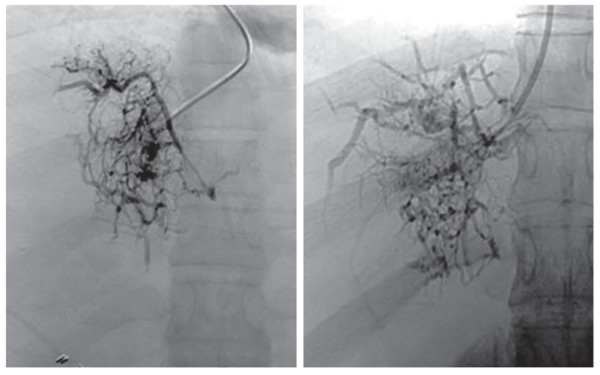
Abdominal venogram

Abdominal ultrasonography showed mild hepatomegaly with diffuse hepatic parenchymal change and also mild splenomegaly (Fig. 2). Endoscopy revealed grade 1 esophageal varices with portal hypertensive gradient (PHG) with fundal varices. Duplex study of portal system revealed features suggestive of early portal hypertension. Venography report revealed dilated and occluding thrombus seen above the hepatic vein level up to right atrium, suggestive of BCS (Fig. 3).

Laboratory investigations concerning thrombophilia revealed that protein S and anticardiolipin antibody levels were in normal limits. Level of protein C was 19 (normal; 70–130).

The patient was finally treated with anticoagulant rivaroxaban after consulting Hematology Department and was discharged from hospital.

## DISCUSSION

Budd–Chiari syndrome is a rare entity among vascular disorder of liver characterized by the obstruction of the hepatic venous outflow tract. Epidemiological data of this rare disorder are limited. It occurs in 1/100000 of the general population.^4^ Etiology-based prevalence of BCS in eastern India showed that idiopathic membranous obstruction and stricture of IVC are the commonest causes of BCS in the eastern part of India. Hepatocellular carcinoma is also a common cause.^5^

The syndrome may be primary when the obstruction is the result of an endoluminal venous lesion like thrombosis or stenosis and secondary causes originates outside the veins. In practice, no identifiable cause is found in up to 70% of patients.^6^ Hepatocellular carcinoma, renal and adrenal adenocarcinoma, renal angiomyolipoma, primary hepatic hemangiosarcoma, sarcoma of the IVC, right atrial myxoma, intrahepatic cholangiocarcinoma, and alveolar hydatid disease have been reported to cause BCS by vascular invasion. The major acquired risk factors include myeloproliferative disease (MPD), APLS, PNH, and Behçet disease. Oral contraceptive use and extreme poverty are major environmental risk factors. Factor V Leiden is the best‑established inherited risk factor for BCS. Recognition of the underlying risk factors is important for appropriate prophylaxis of recurrent thrombosis.^1^

Average age of patients with BCS is 39 years with female predominance. The findings vary from asymptomatic condition to a fulminant picture. Based on differing management and prognosis the disease can be classified into acute, acute on chronic, and chronic group.^1^ In acute or fulminant form the patient presents with rapidly progressive severe upper abdominal pain, mild jaundice, hepatomegaly, vomiting, ascites, elevated liver enzymes, and encephalopathy and usually die within 2 to 3 weeks. In case of total hepatic vein obstruction, delirium, coma, hepatocellular failure, and death within few days occurs. In chronic cases, patient presents with features of cirrhosis along with enlarged tender liver especially caudate lobe, jaundice usually absent or mild and ascites developing over 1 to 6 months with splenomegaly. An asymptomatic form accounting for 15% of cases may have no ascites, hepatomegaly, or abdominal pain.^4^

The condition should be suspected if a patient with a tendency to thrombosis, or with malignant disease in or near the liver, or on oral contraceptives, develops tender hepatomegaly with ascites. An ascitic fluid tap often shows high SAAG exudative fluid but typical of chronic liver disease (CLD) also found. Some supportive biochemical evidences like elevated serum bilirubin within 2 to 3 mg/dL, slight rise in serum alkaline phosphatase level, and serum albumin level reduction are also found in chronic cases.

Ultrasonography with Doppler studies is essential tool for diagnosis. Ultrasonogram features are variable and may include hepatosplenomegaly along with caudate lobe hypertrophy, ascites, hepatic parenchyma in homogenous in chronic cases, and obliteration of hepatic vein, stenosis, spider web vessels, and large collateral vessels. In the acute phase, intraluminal clot may be seen.^7,8^

Doppler ultrasonography shows altered, absent, reversed, turbulent direction of flow in the hepatic vein and retro hepatic IVC. Color Doppler imaging shows abnormalities in the hepatic veins, portal vein, and IVC. In 10% cases portal vein and in 20% cases IVC thrombosis are found.^9^

Hepatic venography may fail or show narrow occluded hepatic veins. Adjacent veins show a tortuous, lace – like spiderweb pattern representing abnormal venous collaterals. The catheter cannot be advanced the usual distance along the hepatic vein and wedges 2 to 12 cm from the diaphragm. Inferior vena cavography establishes the patency of the IVC. The hepatic segment may show side-to-side narrowing due to distortion from the enlarged caudate lobe. Caval pressure measurement is needed.^4^

Computed tomography (CT) scan and magnetic resonance imaging (MRI) are sometimes employed although these methods are not as sensitive. Computed tomography appearances are easily confused with those of hepatic metastases. Magnetic resonance imaging shows absence of normal hepatic venous drainage into the IVC, collateral hepatic veins and signal intensity alterations in the hepatic parenchyma. The caudate lobe can be seen deforming the IVC.

Liver biopsy is nonspecific and shows speckled zone 3 areas can be distinguished from the pale portal areas. Histologically, the picture is of zone 3 congestion.^4^

So all these investigations mentioned above are needed to establish the BCS. Some steps should be taken to evaluate the etiology. A thrombophilia screen must be performed on all patients including protein C, protein S, antithrombin III, factor V Leidin mutation, etc. Myeloproliferative disorder requires screening of the V617F mutation in Janus tyrosine kinase – two gene of granulocytes in blood. Paroxsymal nocturnal hemoglobinuria requires flow cytometry of peripheral blood cells for detection of CD55 and CD59 deficient clones for diagnosis.^1^ A lupus anticoagulant should be done, heart failure, and constrictive pericarditis should be excluded.

Treatment is stepwise starting from medical treatment to liver transplantation as per need. All patients with BCS should receive anticoagulation therapy as soon as possible for an indefinite period. Treatment of the underlying conditions and symptomatic treatment of the complications of portal hypertension are next steps. Angioplasty/stenting of short venous stenoses in symptomatic patients may be applied. Transjugular intrahepatic portosystemic shunt (TIPS) placement in patients not suited for or unresponsive to angioplasty/stenting can be accomplished. The liver transplantation in patients unresponsive to TIPS or with fulminant hepatic failure^2^ can be recommended.

In the last four decades, the prognosis of BCS has improved, with current survival rates of 87, 82 to 93% at 1 year, and 82% at 2 years. Hepatocellular carcinoma developing in BCS patients and polycythemia vera or essential thrombocythemic transformation in myelofibrosis or acute leukemia are the current main concerns in patients with BCS once the initial manifestations of the disease have been recognized and treated.^1^

## CONCLUSION

All prothrombotic conditions must be evaluated while investigating the etiology of BCS, not only latent polycythemia vera and malignancies but also factor V Leiden mutation, protein C, Protein S, and antithrombin III. Also, appropriate antithrombotic and surgical therapies must be performed without delay.
